# Concurrent ecological and evolutionary processes contribute to mutualism breakdown between legumes and rhizobia

**DOI:** 10.1093/ismejo/wrag228

**Published:** 2026-09-17

**Authors:** Sierra L Bedwell, Isabelle M Lakis, Allison R Megow, Angelina E Carpenter, Kevin D Ricks, Christopher J Fields, Jennifer A Lau, Rachel J Whitaker, Katy D Heath

**Affiliations:** Department of Microbiology, University of Illinois Urbana-Champaign, Urbana, IL 61801, United States; Carl R Woese Institute for Genomic Biology, University of Illinois Urbana-Champaign, Urbana, 61801, IL, United States; Department of Microbiology, University of Illinois Urbana-Champaign, Urbana, IL 61801, United States; Carl R Woese Institute for Genomic Biology, University of Illinois Urbana-Champaign, Urbana, 61801, IL, United States; Carl R Woese Institute for Genomic Biology, University of Illinois Urbana-Champaign, Urbana, 61801, IL, United States; Department of Crop Sciences, University of Illinois Urbana-Champaign, Urbana, 61801, IL, United States; Carl R Woese Institute for Genomic Biology, University of Illinois Urbana-Champaign, Urbana, 61801, IL, United States; School of Integrative Biology, University of Illinois Urbana-Champaign, Urbana, IL 61801, United States; Carl R Woese Institute for Genomic Biology, University of Illinois Urbana-Champaign, Urbana, 61801, IL, United States; Department of Natural Resources and Environmental Sciences, University of Illinois Urbana-Champaign, Urbana, IL 61801, United States; Carl R Woese Institute for Genomic Biology, University of Illinois Urbana-Champaign, Urbana, 61801, IL, United States; Roy J. Carver Biotechnology Center, University of Illinois Urbana-Champaign, Urbana, IL 61801, United States; Department of Biology, Indiana University Bloomington, Bloomington, IN, United States; Department of Microbiology, University of Illinois Urbana-Champaign, Urbana, IL 61801, United States; Carl R Woese Institute for Genomic Biology, University of Illinois Urbana-Champaign, Urbana, 61801, IL, United States; Carl R Woese Institute for Genomic Biology, University of Illinois Urbana-Champaign, Urbana, 61801, IL, United States; Department of Plant Biology, University of Illinois Urbana-Champaign, Urbana, IL 61801, United States

**Keywords:** ecology, evolution, mutualism, mutualism breakdown, nitrogen fixation, rhizobia

## Abstract

Though they jointly shape community responses to environmental perturbations, ecology and evolution are often examined separately, even in microorganisms where both occur over short timescales. Here, we examine ecological and evolutionary responses to 33 years of nitrogen fertilization using the legume–rhizobium mutualism. Pairing a manipulative inoculation study with full-length 16S rRNA gene amplicon sequencing and structural equation modeling allows us to synthesize across biological scales: whole bacterial community, genus *Rhizobium, Rhizobium* amplicon sequence variants (ASVs), and symbiosis plasmids. Clover’s preferred partner decreases in N-addition soils, limiting host growth, while a diverse and largely uncharacterized *Rhizobium* community increases. This ecological change is compounded by a concurrent evolutionary degradation of symbiont partner quality via changing frequencies of symbiotic plasmids. Ecological (rarer symbionts) and evolutionary (inferior symbionts) processes each accounted for roughly half of this loss of host benefit, revealing that ecology and evolution jointly shape mutualism breakdown over the short timescales typical of microbial systems.

## Introduction

Plant–microbe mutualisms are some of the most important symbioses on Earth, altering biogeochemical cycles [1, 2], promoting plant productivity [3, 4], and protecting hosts from stress and enemies [5–7]. These mutually beneficial interactions between species are fundamental to every ecosystem and yet may be unstable [8–12]. Under environmental change, mutualisms may break down, with partners potentially evolving to parasitize their host, switching to new hosts, or adopting a free-living lifestyle [12–14]. Thus, understanding why and how mutualisms break down will be essential to maintaining them in the face of a changing world, and tractable mutualisms offer ideal models with which to examine the interplay of ecology and evolution in this process.

Mutualism breakdown has been observed in multiple systems due to either ecological or evolutionary changes. Ecological changes such as community-level shifts in composition can drive decline; e.g. multiple studies have found that invading ants can disrupt the existing mutualism between African ants and acacia trees [15, 16]. Additionally, breakdown through evolutionary mechanisms such as population-level shifts in allele frequency has been observed in diverse systems such as corals and pollinator wasps [12, 17–19]. Though models have predicted that mutualisms can break down through concurrent ecological and evolutionary processes [20, 21], this has been difficult to measure in natural systems. In macroorganisms, generation times are slow enough that incorporating evolutionary processes into short-term experimental systems is difficult, while in microbes, ecology and evolution occur on comparable timescales, yet cannot easily be differentiated [22, 23].

In microbial communities, allele-frequency shifts within populations of single microbial species may occur alongside community composition shifts at broader taxonomic scales [23]. Yet, in microbes, these processes have often been examined using different techniques. For example, population genomics and classical experimental evolution provide high resolution of allelic shifts within species, but: (i) require isolates to be culturable [24] and (ii) may miss key dynamics occurring between species in a more complex community [25, 26]. In contrast, standard amplicon sequencing (e.g. the V4 region of the 16S rRNA gene) can capture a large portion of unculturable diversity in complex microbial communities, but has been limited by amplicon length and sequencing depth, thus restricting fine-scale resolution needed to move beyond community shifts at the broad scale (phylum, order, and family) [27, 28]. Additionally, microbial populations and communities are interconnected by the movement of genes within and between taxa through horizontal gene transfer (HGT) [29, 30]. For microbial mutualists with mobile symbiosis genes [31, 32], this movement may accelerate both the emergence and breakdown of mutualism [33–35], as well as potentially blurring the lines between ecological and evolutionary processes [23, 36].

The legume–rhizobium mutualism is an ideal model to examine the ecological and evolutionary dynamics of mutualism breakdown because it consists of a taxonomically limited set of focal symbionts within progressively more complex communities, from the internal structures of the host plant to the hyperdiverse soil environment. In the resource mutualism between leguminous plants and nitrogen-fixing rhizobia, rhizobial cells living in soil form nodules on legume roots and therein fix atmospheric dinitrogen gas into plant-available forms of nitrogen (N) in exchange for carbon from the plant host [4, 37]. In the *Trifolium* (clover)*–Rhizobium* symbiosis, and many others, the mutualism relies on the presence of symbiosis genes carried on mobile genetic elements (MGEs), often plasmids (pSyms) or integrative conjugative elements (symICEs) [32, 34].

Previous work in this system has shown that mutualism can break down under long-term fertilizer (N) addition, and culture-dependent studies suggested an evolutionary explanation by identifying genetic changes located within the pSyms that differentiated isolates from fertilized versus unfertilized populations [38, 39]. In fact, isolates collected from N-addition soils often contained evolutionarily distinct pSyms that conferred worse partner quality [40]. However, when these genetic effects were isolated using single inoculation experiments, the impacts on host growth were much smaller compared to those estimated when plants were inoculated with whole soil from those same plots [38], suggesting that ecological changes in rhizobial community composition and/or abundances, in addition to rhizobium evolution, also play a key role in mutualism breakdown between clover and rhizobia under long-term N-addition [38–40].

Addressing potential ecological changes that may have taken place in these communities under long-term fertilization requires a much deeper understanding of this extremely diverse group (the genus *Rhizobium* as well as the larger family *Rhizobiaceae*). This group has posed taxonomic challenges—both because naming conventions for plant symbionts often predate modern microbial taxonomy [41] and because, until recently, there were insufficient full reference genomes distributed across the entire family with which to discriminate chromosomal genera [42, 43]. The family *Rhizobiaceae* consists of at least 38 diverse genera and nearly 300 species, which are linked by extensive HGT and plasmid exchange [42, 44, 45]. Even the delineation of genus and species is debated, with comparative genomics revealing that *Rhizobium leguminosarum* itself actually comprises a larger group of at least 18 closely related genospecies, the *R. leguminosarum* species complex (Rlc) [43]. These genospecies, only some of which have received formal species names despite clearly falling outside species boundaries, can have average nucleotide identity (ANI) relatedness of as low as 92%, yet are still distinctly separated from other members of the *Rhizobium* genus such as *Rhizobium etli* [43, 46].

Posing an additional challenge to characterization, even this vast rhizobial diversity comes mainly from nodule isolates, which may have experienced significant host filtering and may not represent overall community diversity [47–49]. For example, our previous culture collection of 63 isolates only captured 2 genospecies (gsE and gsB [38, 40])—only 11% of the total *R. leguminosarum* genospecies diversity identified in clover nodules worldwide [43]. Culture-dependent work has suggested that rhizobia in soil are rare compared to other bacterial taxa, yet may be diverse [50, 51]. Thus, making sense of rhizobial communities using standard culture-independent methods is challenging, because short-read amplicon sequencing often lacks both the resolution to differentiate disparate genera within the family [43, 44] and the depth to capture rare taxa in hyperdiverse communities. It is clear that understanding any ecological drivers of breakdown within this mutualism requires high-resolution, culture-independent data that can characterize rhizobial diversity in and out of nodules.

Here, we leverage a long-term N-fertilization experiment with high-throughput, long-read amplicon sequencing of microbial communities from soil, rhizosphere, and nodules from a common garden greenhouse inoculation experiment to determine how 33 years of N-fertilization has affected legume-associated microbial mutualists at multiple scales. First, we examined the diversity of microbial communities across compartments. Then, we focused on ecological community shifts of rhizobial taxa, as well as evolutionary changes in a conserved pSym gene, *nodA*. We used piecewise structural equation modeling (piecewise SEM) to model how these concurrent ecological and evolutionary forces contribute to a decline in mutualistic partner quality and thus clover host biomass. Finally, we examine the phylogenetic diversity of the whole rhizobial community throughout plant compartments and between N-addition and control to determine how this important symbiont community responds to environmental change. In this work, we unpack how the microbial community, across all levels, is impacted by N-fertilization across plant compartments, from field soil, to greenhouse soil, to plant rhizospheres, to symbiotic nodules.

## Materials and methods

Full details for all experiments, analyses, and packages are provided in Supplementary Data. We summarize the key approaches below.

### Study site and soil collection

Our inoculum comes from the successional long-term N-fertilization experiment (the T7 untilled microplots) at the Kellogg Biological Station Long-Term Ecological Research Site (KBS LTER) in Michigan, USA. This experiment includes 6 pairs of 25 m^2^ plots: with 1 plot in each pair fertilized since 1989 with agriculturally relevant levels of nitrogen and the other remaining unfertilized [52]. We sampled field soil in July 2022 after 33 years of N-fertilization; taking 48 soil cores in total (4 per plot × 6 plots per N treatment × 2 N treatments).

### Greenhouse experiment

We inoculated 2 ml slurries from each core onto separate pots, containing a single 1-week old seedling of either one of two clover species—*Trifolium hybridum* and *Trifolium repens*. Inoculants and clover species were randomly assigned to pots in each of three replicate blocks. Plants were never provided exogenous nitrogen.

Each block contained 126 plant pots including 30 controls. Plants were harvested after 8 weeks. We separated aboveground and belowground biomass and additionally collected one 15 mL tube of bulk soil from each pot. In the lab, we weighed aboveground biomass for all plants. For sequencing, we separately processed bulk soil, rhizosphere, and nodule samples.

### PacBio full-length 16S rRNA gene and *nodA* gene sequencing and sequence processing

We sequenced the full-length 16S rRNA gene from source soil, bulk soil, rhizosphere, and nodule samples, and *nodA* from nodules. Library construction and sequencing on the Revio System (Pacific Biosciences, CA, USA) were performed at the Roy J. Carver Biotechnology Center, University of Illinois. Initial sequence processing was completed by the High Performance Computing Group (HPCBio) at the University of Illinois. To verify that absolute and relative abundance were correlated, we sequenced the 16S rRNA gene of ten bulk soil samples with a commercial spike-in and quantified absolute cell counts (Fig. S1).

After quality filtering and *post hoc* chimera removal, we retained 7761 ASVs across 389 genera. Saturating rarefaction curves indicated that even after filtering, we captured the majority of diversity present (Fig. S2).

### *Rhizobium* classification

We used the SILVA delineation of the genus *Allorhizobium-Neorhizobium-Pararhizobium-Rhizobium* (referred to hereafter as the ANPR group) for initial classification (172 ASVs). To classify these ASVs below the genus level and account for the extreme polyphyletic nature of the *Rhizobium* genus, we used the updated *Rhizobiaceae* framework [42]*,* placing references alongside ASVs in an IQ-TREE phylogeny. The full-length 16S rRNA gene resolves the clade comprising *Rhizobium sensu stricto* (*leguminosarum*–*etli*), *Martinezella* (*tropici*–*rhizogenes*), and *Arminella* (*tubonense*–*tumorigenes*) from more distant genera, but does not reliably differentiate these three genera from one another; we therefore refer to this clade as *Rhizobium* sensu lato (s.l.) (44 ASVs). To more specifically classify genospecies members of the *R. leguminosarum* species complex (*Rlc*), we used the genospecies framework [43].

### Statistical analyses

All analyses were conducted in R 4.5.1 (2025-6-13). We tested community composition (whole microbial community, ANPR, and *Rhizobium* s.l.) against N-treatment, plant compartment, and plant species using PERMANOVA on Bray–Curtis dissimilarities of relative ASV abundances, visualized with dbRDA.

Effects of N addition on nodule endophyte diversity, ASV-E relative abundance, and *Rhizobium* s.l. community metrics (relative abundance, number of ASVs, evenness, Shannon Index) were tested with linear mixed-effects models including plot as a random effect. We used Fisher’s exact tests to compare the proportion of ANPR-dominated nodules and the frequency of nodulation between treatments, a Wilcoxon rank-sum test to compare shoot biomass between subtype C pSym-dominated nodules and all others, and a Type III ANOVA to compare plant biomass between treatments. Differential abundance of *Rhizobium* s.l. ASVs and *nodA* alleles was assessed with DESeq2.

To partition variance in plant biomass attributable to ecological versus evolutionary processes, we fit a two-part piecewise structural equation model (SEM) reflecting the sequential structure of abundance → nodulation → symbiont quality → biomass: a logistic regression of source soil ASV-E relative abundance on binary nodulation, and a linear model predicting biomass from nodulation, pSym subtype C dominance, and direct ASV-E abundance. We compared ecological (indirect × direct) and evolutionary effect sizes by bootstrapping.

### Usage of large language models

To generate and edit code, as well as to edit text for grammar, the large language models (LLMs) ChatGPT (OpenAI, CA, USA) and Claude (Anthropic, CA, USA) were utilized. The authors then reviewed and edited all LLM-produced content and take full responsibility for the content of this publication.

## Results

### Microbial community composition was strongly structured by plant compartment

As expected, the largest source of variation in microbial community composition was plant compartment [i.e. source soil (directly from the field), bulk soil (from pots in the greenhouse), rhizosphere (directly in contact with plants), or nodule], explaining 32% of total variance (Table S1A and B and Fig. S3). Inoculum source (whether the microbial community came from control versus N-addition soil slurries) and the compartment x inoculum interaction contributed 1.2% and 3.7% of the total variance overall, respectively. However, the effects of inoculum source varied considerably across compartments; inoculum source effects were strongest in the field source soil (31.8% of variance), weaker in the greenhouse bulk soil and rhizosphere (3.4% and 3.5% of variance, respectively), and not significant in the clover nodule (Table S1B). Whereas the source (field) and greenhouse bulk soil were dominated by common soil genera [e.g. *Bacillus*, *Nocardioides,* Supplementary Data (SD) 1–3], the rhizosphere and nodules harbored plant-associated communities including the symbiont genera *Allorhizobium-Neorhizobium-Pararhizobium-Rhizobium* (ANPR) (SD1–3).

### Nodules are diverse yet dominated by a few ANPR ASVs

Because nodule taxa might be important drivers of clover health, we first asked which genera and ASVs dominated the nodule compartment. Nine different genera had mean relative abundances of above 1% in nodules (Fig. 1A), including many known non-rhizobial endophytes (NREs) such as *Cupriavidus, Pseudomonas,* and *Roseateles.* Overall, 20% of nodules were dominated by one of these non-rhizobium taxa (Fig. S4), and the diversity of NREs did not differ between plants inoculated with fertilized or unfertilized soil (*P* = .25). Nonetheless, ANPR was the most abundant genus in clover nodules, with a mean relative abundance of 58%, a pattern which also did not differ between fertilized and unfertilized inoculum sources (Fisher’s exact *P* value = .1674). Though we had limited power to interrogate the impacts of individual NREs on plant biomass, we found no difference in plant biomass between samples dominated by ANPR versus other NREs, likely because even when nodules were not dominated by ANPR, half (11/22) maintained at least a 25% relative abundance of ANPR (results not shown).

**Figure 1:**
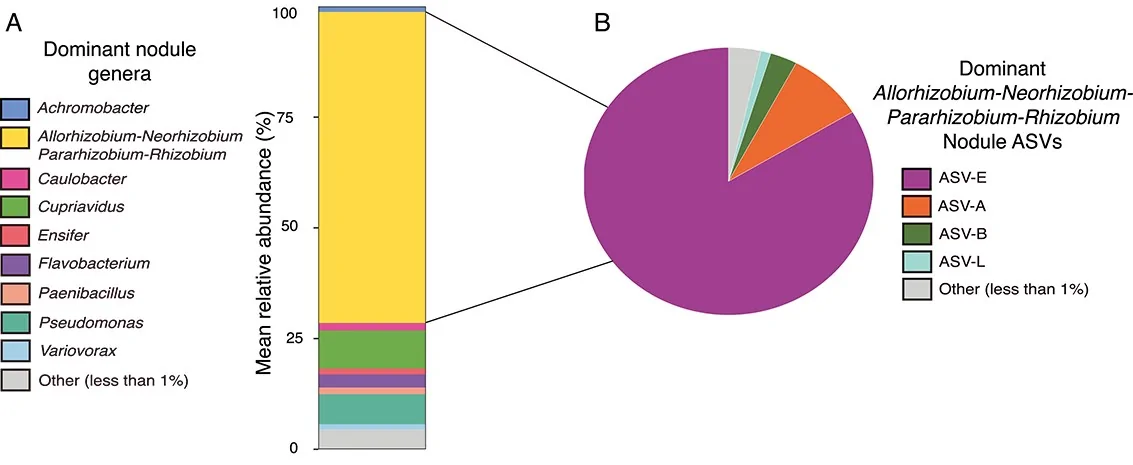
Nodules are diverse yet dominated by a few ANPR ASVs. (A) Composition of nodules at the genus level across nitrogen and control. (B) Composition of *Rhizobium* ASVs across nitrogen and control. For both, stacked bars represent the mean relative abundance of genera or ASVs between nodule samples. Each individual sample (comprised of 10 nodules per plant homogenized) was relativized, then all relative abundances were averaged. All genera/ASVs comprising less than 1% of reads in individual nodule samples were collapsed to “Other”.

Among the ANPR ASVs in the nodules, only four maintained an abundance of more than 1% (Fig. 1B). To identify these ASVs, we BLASTed them against a current *Rhizobiaceae* database (SD8-9) [42]. The most abundant (ASV-E) was identical to the 16S rRNA gene sequence for *R. leguminosarum sensu stricto* (gsE), which was previously found to dominate clover nodules in this environment [40]; this sequence also matches several other genospecies (such as *Rhizobium brockwellii*/gsA*, Rhizobium laguerreae*/gsR*, Rhizobium ruizarguesonis*/gsC) and the more distant *Rhizobium hidalgonense*—none of which have been isolated from nodules from KBS*.* The ANPR taxon ASV-E was dominant within nodules (Fig. 1B) and was the second most ubiquitous ASV in the whole microbial community, present in 93% of all samples (SD4–6). Three other ANPR ASVs had mean relative abundances above 1% in nodules: ASV-B (an ASV which shared 100% identity to *Rhizobium johnstonii* (gsB) and *Rhizobium redzepovicii*), ASV-A (100% identical to *Agrobacterium arsenijevicii* and *Agrobacterium tumefaciens*) and ASV-L (which was one single nucleotide polymorphism (SNP) different from *Rhizobium altiplani*).

### ASV-E is both less abundant and lower quality under N-addition

Given the dominance of ASV-E, as well as previous work implicating gsE’s role in clover health in this system [38–40], we next examined how the relative abundance of ASV-E (relative to other ANPR ASVs) changed between N-addition and control inocula, and how these shifts related to plant growth in our inoculation study. We found that ASV-E was ~3-fold less abundant in the N-addition source (field) soils (*P* < .0001, Fig. 2A, Table S2) and ~1.7-fold less abundant in bulk soil of plants inoculated with N-addition field soil slurries (*P* < .05, Fig. 2A, Table S2), though there was no difference within the rhizosphere or nodule compartments.

**Figure 2:**
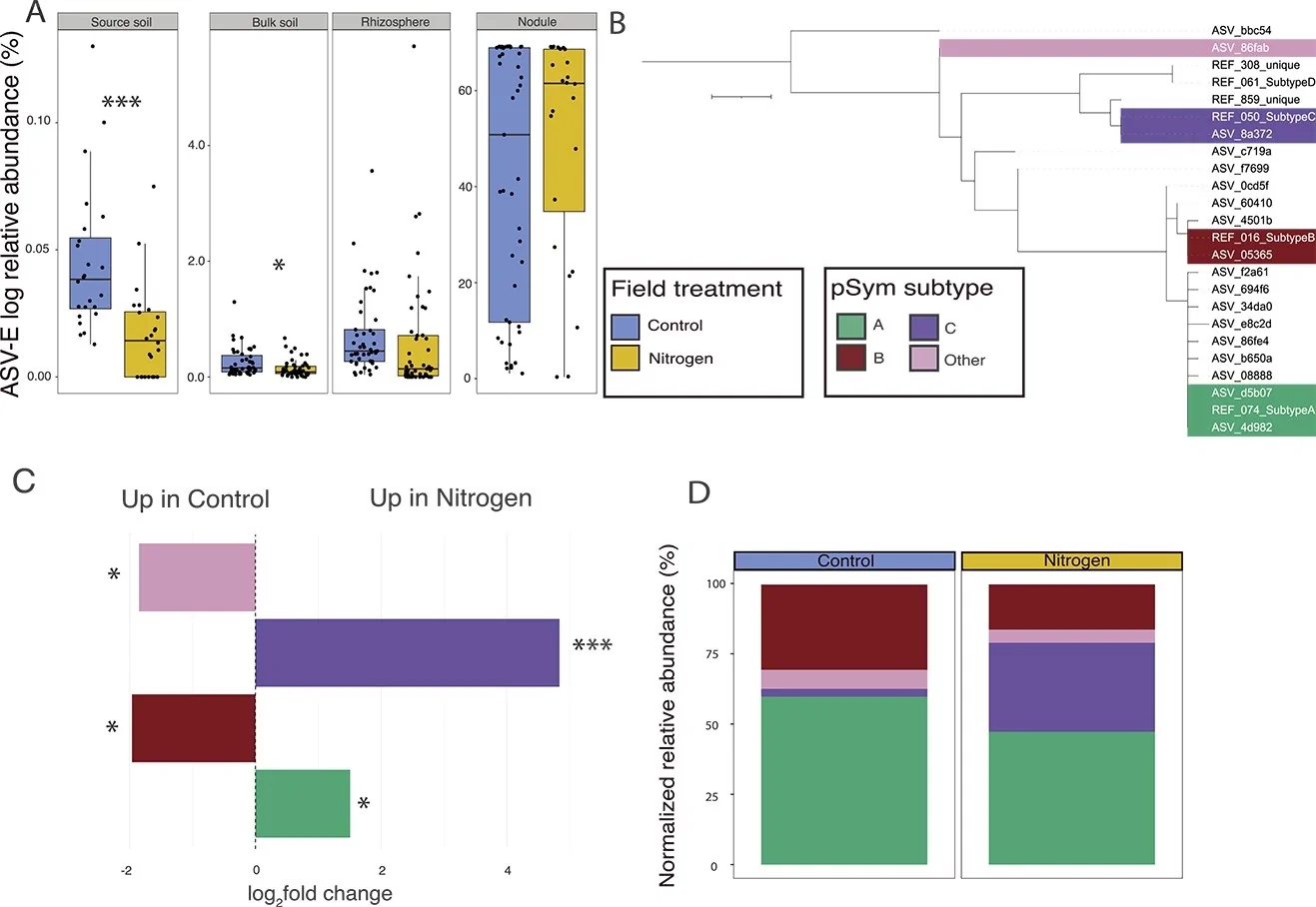
Under long-term N-addition, ASV-E is less abundant, and nodules are dominated by low-quality pSym subtype C. (A) Relative abundance of ASV-E is significantly lower (*P* < .05) in nitrogen field source and bulk soil. Significance determined using linear mixed model considering nitrogen treatment and plot (nested within N-treatment). Compartments were modeled individually due to the large fold-changes in relative abundance between compartments which masked nitrogen effects. (B) *nodA* phylogenetic tree. Reference sequences from our recent study [40]*.* Tree computed using MAFFT/IQ-TREE and visualized in iTOL. (C) log_2_fc of *nodA*. Significance determined using DESeq2, *P* for subtype C = 1.0 × 10^−11^, *P* for “other” = 2.5 × 10^−2^. (D) *nodA* stacked bar plot. Stacked bar plot depicts relative abundance normalized by sample number (Control = 45, Nitrogen = 24). Within B, C, and D, the two subtype A ASVs contained one SNP toward the end of their sequenced region, which was ultimately trimmed out so all ASVs would be the same length as the reference genes; thus, the reads of these ASVs were combined in all analyses done on subtype A.

We also quantified the relative frequencies of previously characterized [40] high-quality and low-quality pSym subtypes in nodules from N-addition and control inocula using a marker of pSym type (*nodA*). An ASV matching to low-quality pSym subtype C was >28-fold enriched in nodules formed by plants inoculated with N-addition field soil slurries (Fig. 2B and C). Additionally, we found three more dominant *nodA* types (together, all four representing over 98% of sequencing reads). One ASV matched 100% to high-quality *nodA* subtype B (Fig. 2B), whereas two additional ASVs, one very common and the other extremely rare, both matched 100% to high-quality *nodA* subtype A. Additionally, a third abundant type (ASV_86f) has not yet been described, yet was more closely related to our *R. leguminosarum nodA* sequences than our natural outgroup (a rare *Rhizobium nodA* ASV with over 20 SNPs to all other ASVs; Fig. 2B). This ASV, though the rarest of the dominant four, was slightly enriched in nodules from plants inoculated with control (versus N-addition) soil slurries (Fig. 2C and D).

These observed changes in soil microbial communities were associated with differences in plant growth. Plants inoculated with soil slurries from long-term N-fertilization treatments produced 55% less biomass (Fig. 3A). We fit a two-part piecewise SEM across all 48 source soil cores to partition the standardized effects of ecology (the direct and indirect, nodulation-mediated effects of ASV-E relative abundance on biomass) from evolution (the direct effect of subtype C pSym dominance on biomass). The ecological and evolutionary impacts did not differ in magnitude (bootstrap *P =* .33): the total standardized ecological effect on biomass (direct + nodulation-mediated indirect paths) was 0.44 SD, whereas the standardized evolutionary effect of the pSym subtype on biomass was −0.25 SD (so plants dominated by subtype C were smaller than those with other pSym types) (*P* = .069) (Fig. 3B, Table S3). Within the ecological path, both legs of the indirect path through nodulation were significant (Fig. 3B, Table S3), and the indirect path (total effect size = 0.33) contributed more than the direct path (effect size = 0.11, *P* = .441)—suggesting that the majority of ASV-E’s ecological impact on plant biomass flows through nodule formation (Fig. 3C and D).

**Figure 3:**
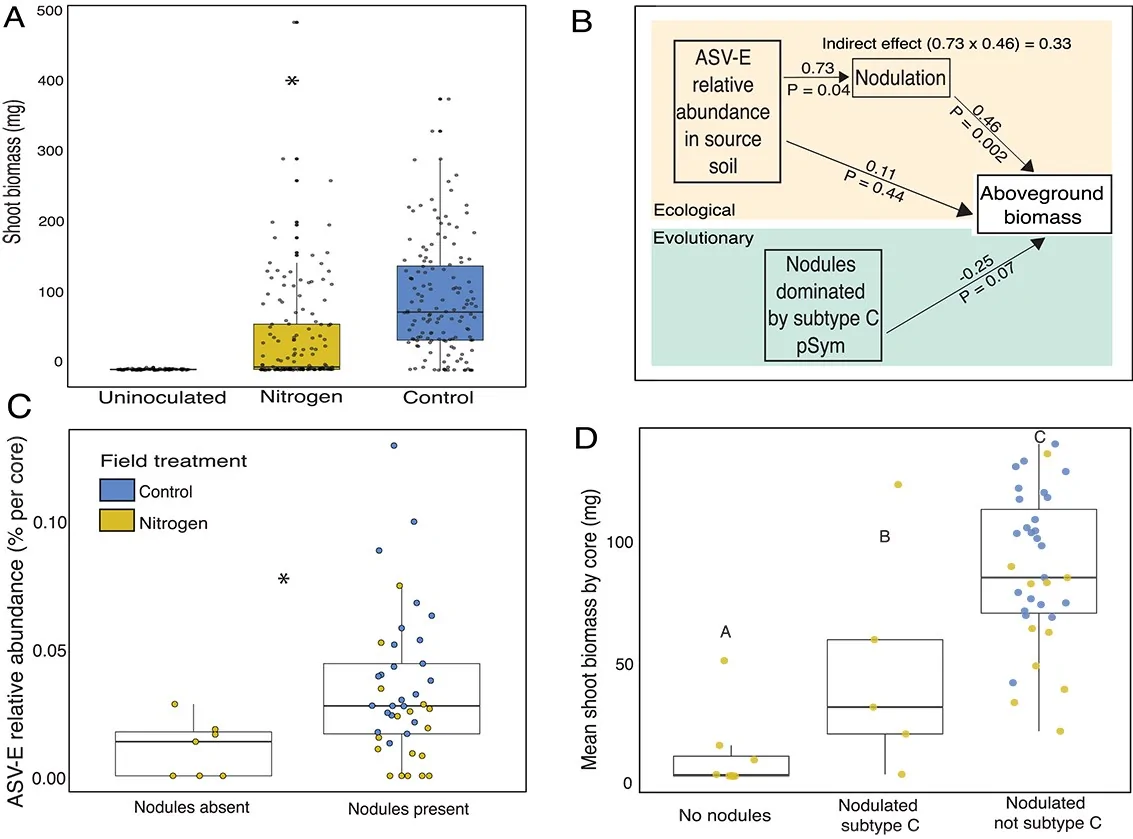
ASV-E relative abundance and pSym type predict plant health. (A) N-addition reduces clover aboveground biomass. Aboveground plant biomass is significantly lower when plants are grown in soil from N-deposition plots (*F*_1,284_ = 22.0, *P <* .01, *N* = 288). (B) Ecological and evolutionary pathways both contribute to changes in aboveground biomass. Results from piecewise SEM. The ecological effect takes into account both the direct impact of ASV-E in source soil on biomass as well as the indirect effect of ASV-E → nodulation → biomass, the evolutionary impact takes into account the direct effect of nodules being dominated by the subtype C pSym on biomass. Two-sided *P* value for the difference in magnitude between ecological and evolutionary impacts = 0.33. Full coefficients found in Table S3. (C) Relative abundance of ASV-E in the source soil predicts whether clover nodulates. *N* = 48. Significance was determined using a Wilcoxon rank-sum test (Mann–Whitney U test), 0.017. (D) Regardless of N-fertilization, when pSym subtype C is dominant in nodules, plant biomass is significantly lower. Dominance was defined as over 50% of the relative abundance of *nodA* within a nodule being comprised of pSym subtype C. Kruskal–Wallis chi-squared = 20.8, df = 2, *P* = 3 × 10^−5^. Letters indicate groups that differ at BH-adjusted *P* < .05 (pairwise Wilcoxon rank-sum). *P* between A and B = 0.034, *P* between B and C = 0.034, *P* between A and C = 7.9 × 10^−7^.

### Most ANPR ASVs are not *Rhizobium*, and few are compartment specialists

Next, to examine rhizobial community composition and changes under N-addition more deeply, we classified the 172 ANPR ASVs beyond the SILVA delineation (*Allorhizobium-Neorhizobium-Pararhizobium-Rhizobium*). We constructed a phylogeny by using up-to-date references across the family *Rhizobiaceae* (SD7–8) [42]*.* From this tree, we were able to identify 44 ASVs (26%) in the genus *Rhizobium* s.l., (3 genera formerly known as *Rhizobium* and just recently split) [42] which cleanly separated from the rest of the genera within the family (Fig. 4). We also observed that, contrary to our expectations by filtering our initial selection to the ANPR genera, the majority of our ASVs (54%) did not group with any of the four ANPR genera, and instead were most closely related to ~10 additional genera within the family *Rhizobiaceae* (Fig. 4). Though many of the genera we observed have only been defined after the initial identification database we used was released (such as *Onobrychidicola*, described in 2022, of which we had 18 ASVs), we identified one ASV which, at least by 16S rRNA, was phylogenetically similar to *Shinella*, an entirely separate genus in SILVA that we had explicitly filtered out in our analyses (Fig. 4). Another ASV, which despite passing all our filtering cutoffs, had less than 95% identity to every reference and a branch length 300 times longer than its neighbors, and was subsequently removed from our phylogenetic tree. Additionally, the majority of these ASVs (80%) had no perfect matches to known *Rhizobiaceae* and likely represent a significant expansion of known diversity in the family. Both the larger ANPR group and the true *Rhizobium* s.l. genus were compartment generalists, with 70% (122/172) present in both plant-associated (rhizosphere/nodules) and soil (bulk/source) environments. Additionally, though nodule presence was not common (only 27% of ANPR ASVs were seen in nodules), it was not phylogenetically restricted to the true *Rhizobium* genus (Fig. 4).

**Figure 4:**
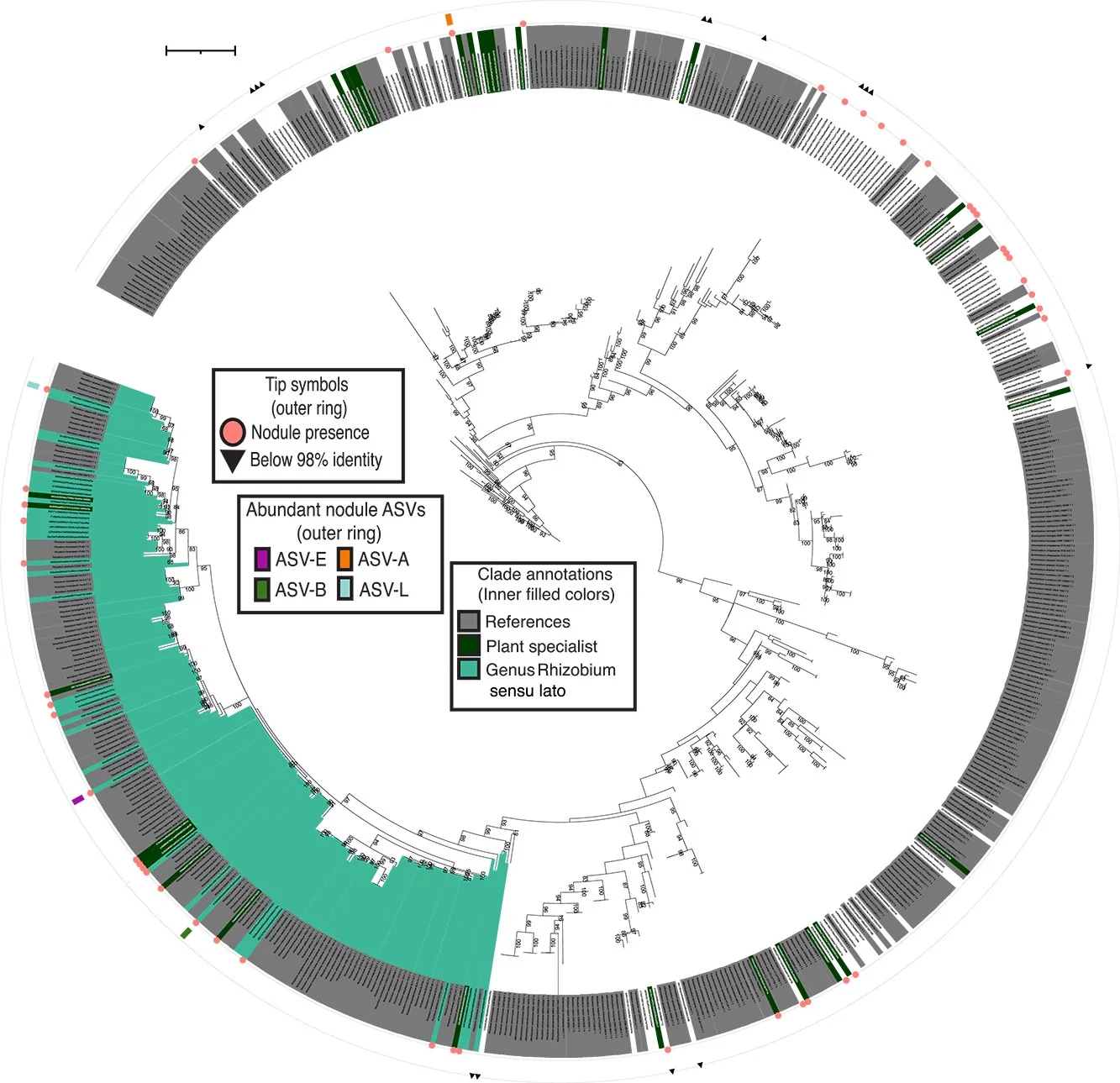
ASVs filtered to Allo-Neo-Para-Rhizobium span the entire family *Rhizobiaceae* and contain 44 *Rhizobium s.l.* ASVs. Phylogenetic tree of 171 *Allorhizobium-Neorhizobium-Pararhizobium-Rhizobium* ASVs (one ASV was removed due to extremely low phylogenetic relatedness to everything else) and 245 reference sequences (labeled in gray at the tip level) from the family *Rhizobiaceae*. Tree is rooted on the genus *Mesorhizobium*. The three genera classified as *Rhizobium* s.l. are not resolvable at the level of the 16S rRNA gene and are colored at the clade-level. Plant specialist ASVs (only found in rhizosphere and/or nodules) are labeled in green at the tips. Any presence in nodules is recorded as a circle on the outside. ASVs that never have matches above 98% identity are labeled on the outside with triangles. Abundant nodule ASVs (above 1% relative abundance overall) are labeled on the outside as rectangles. Genera within *Rhizobiaceae* are labeled in colours within clades (and extending to branches for ASVs that are not plant specialists or references). Genus delineations, including genus *Rhizobium*, were defined from previous work. Scale bar represents 0.1. A tree with all genera labeled can be found on iTOL; link in the Supplementary methods.

### Genus-level aggregation masks ASV-level responses to N-addition

Using this refined taxonomy, we focused on the *Rhizobium* s.l. genus to understand whether this genus responded to N-addition in a similar manner to ASV-E. Unlike ASV-E, the relative abundance of the entire *Rhizobium* genus did not decrease in response to nitrogen in source soil, bulk soil, or rhizosphere and actually increased in N-addition nodule samples (Fig. 5A). Similarly, N-addition had little effect on the Shannon Index of rhizobia (Fig. 5B), though, similarly to the whole microbial community, diversity decreased strongly across plant compartments: the Shannon Index of nodules was 15-fold lower (Shannon Index ~0.1) than that of the source soil (Shannon Index ~1.5) (Fig. 5B, Table S4B), with evenness and richness showing similar patterns (Fig. 5C and D).

**Figure 5:**
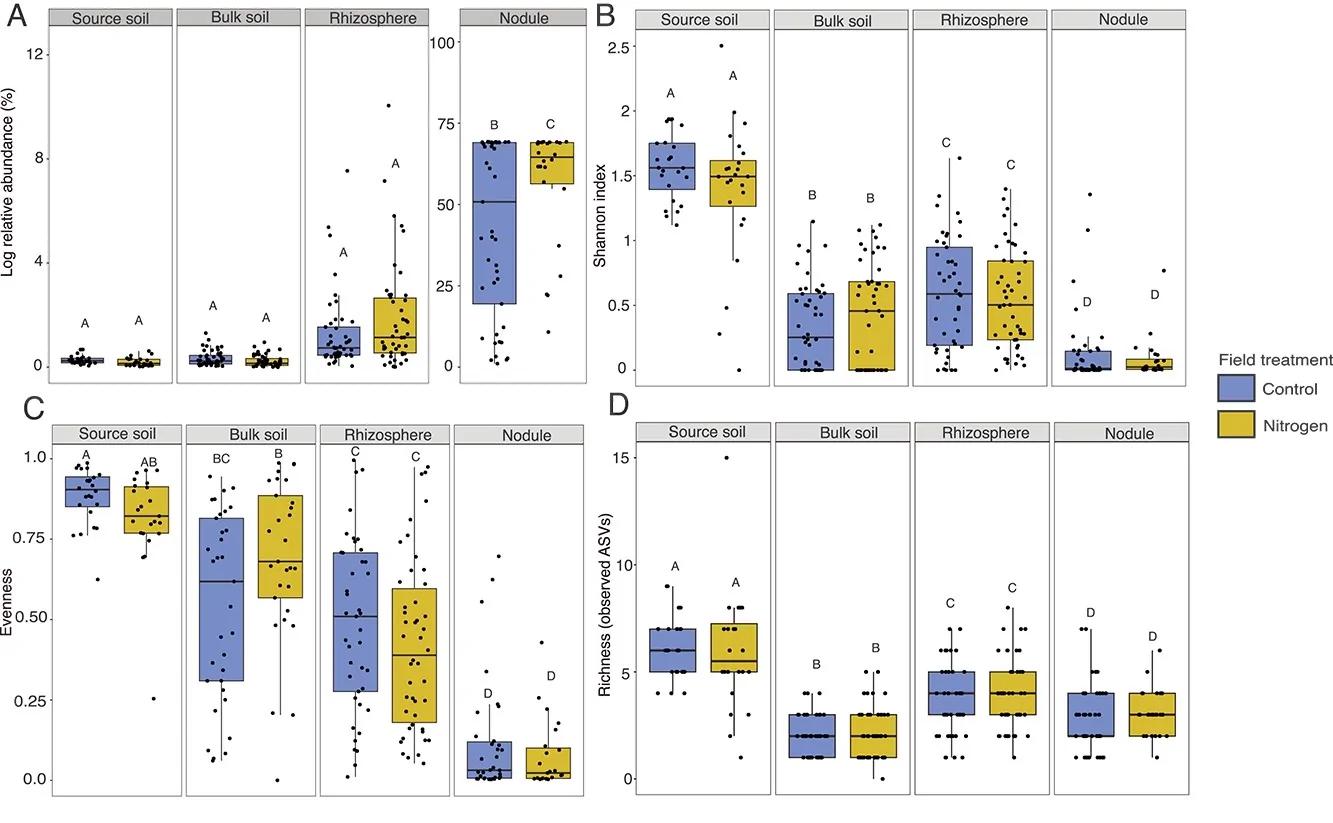
*Rhizobium* genus relative abundance increases (A) while *Rhizobium* species diversity and evenness decreases (B, C) within plants compared to soil environments. These shifts in *Rhizobium* diversity from soil to plant compartments are largely due to increased likelihood of low abundance *Rhizobium* taxa within source soil and rhizosphere (D). (A) Log relative abundance of genus *Rhizobium* increases within plants. *P <* .05. Significance determined by linear mixed-model and Tukey’s *post hoc* test. One upper outlier in the bulk soil (relative abundance 0.29) was removed. Though it was not a statistical outlier, its presence obscured abundance patterns. Letters represent Tukey’s HSD on all 8 Nitrogen x Compartment means (28 comparisons). (B) Shannon Index of genus *Rhizobium* significantly decreases within plants. Significance determined by linear mixed-model and Tukey’s *post hoc* test. (C) Evenness of genus *Rhizobium* significantly decreases within plants. *P <* .05. Significance determined by linear mixed-model and Tukey’s *post hoc* test. Letters represent Tukey’s HSD on all 8 Nitrogen x Compartment means (28 comparisons); 55 values of evenness values were NA, this was because the richness of corresponding samples was 1 and so evenness could not be computed. (D) Low abundance *Rhizobium* significantly increase richness (observed ASVs) in source soil and rhizosphere, *P <* .05. Significance determined by linear mixed-model and Tukey’s *post hoc* test.

The relative abundance of *Rhizobium* did not change in response to N, but we did observe significant shifts in community composition within this genus in source soil, bulk soil, and rhizosphere compartments (nodules were marginal; *P =* .07, Tables S5 and S6, Figs S5 and S6). In all compartments, most ASVs were shared between N-addition and control (Fig. 6A), indicating that ASVs were largely generalists rather than being exclusive to one environment. Nevertheless, 21% of all rhizobial ASVs changed in relative abundance between N-addition and control; some increased, while others decreased (Fig. 6B, SD9–10). Four ASVs with the largest increases under N-addition including ASV-B (one of our top nodule ASVs, discussed above), which is significantly more abundant in N-addition in all compartments though only dominates the nodules of two plants (Fig. 6B and C). Second, ASV-U (465f73b2a423c62bd060565910acbc79, right next to *Rhizobium rhizogenes*; Fig. 4) was never found in the nodules but was significantly more abundant in the three other compartments (Fig. 6B and C). Finally, two more ASVs were rare and found only in the source soil (ASV-T: 97f93469eabe0ba1d6933d08c02f1e9d and ASV-M: 9e84fdaea54f2ca70cf83a3afb04af2a; Fig. 6B and C, SD9).

**Figure 6:**
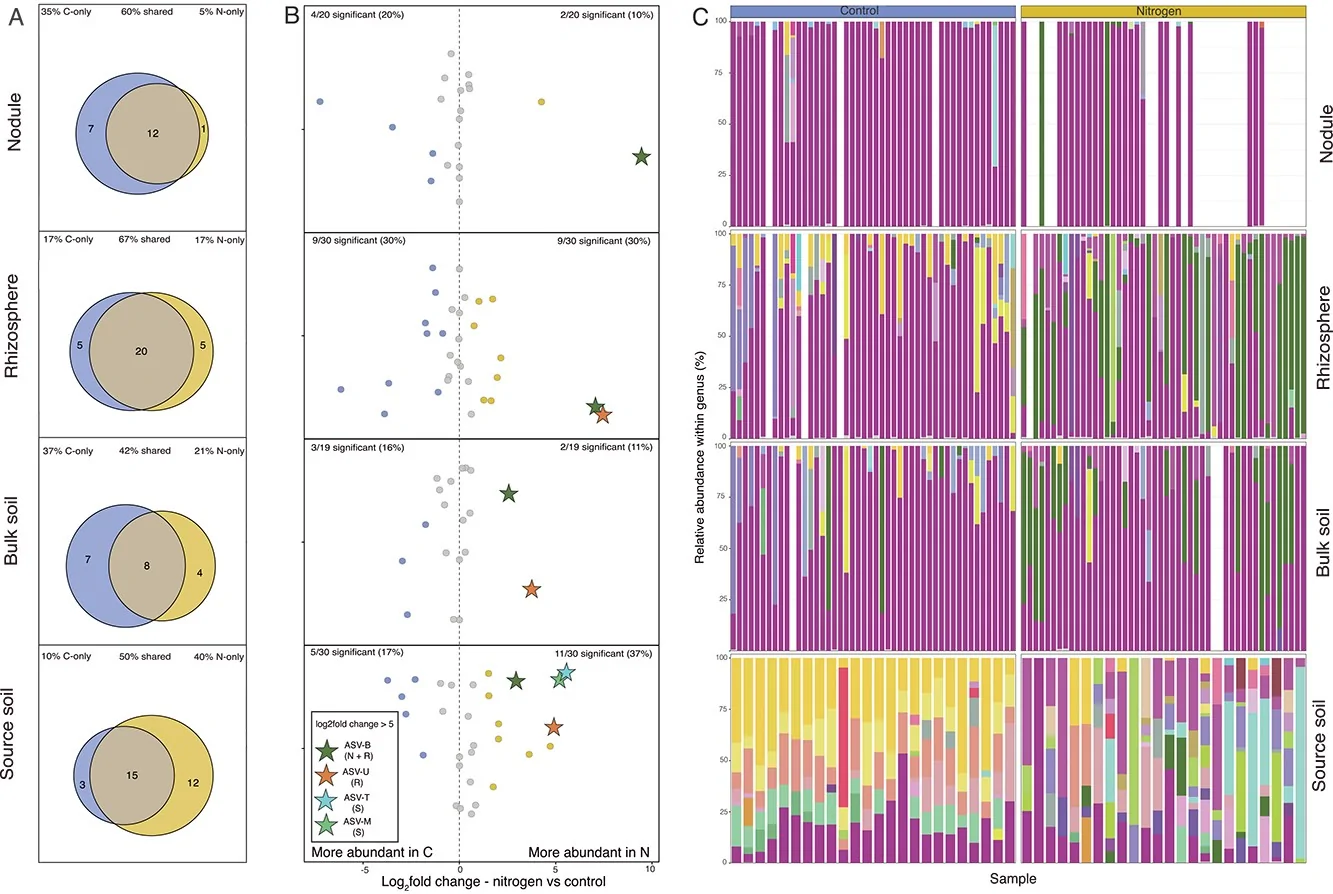
The majority of *Rhizobium* ASVs across compartments are shared between N-addition and control (A), despite strong changes in relative abundance (B, C). (A) The majority of *Rhizobium* ASVs are shared between N and C**.** Compartment-level Venn diagrams depicting whether *Rhizobium* ASVs are unique to N, to C, or are shared between both environments. (B) Individual *Rhizobium* ASVs respond strongly to N-fertilization within compartments. Log_2_fold change calculated for 44 *Rhizobium* ASVs across 4 compartments between nitrogen and control environments. Colored circles (often towards the outside extremes) represent significant ASVs (*P* < .05), gray circles (often toward the middle) represent nonsignificant ASVs *(P >* .05). Four ASVs with log_2_fold changes greater than 5 in at least one compartment are highlighted with stars instead of dots in every compartment they are present in. Points are jittered for clarity along a *Y* axis. (C) Species-level diversity of *Rhizobium* is high but varies strongly between N-addition and control. Stacked bar plots of *Rhizobium* ASVs organized by plant compartment. Colors represent unique ASVs; color assignments are consistent within panels. ASV relative abundance is normalized within genus. Bars arranged left to right by sample, with the same plant samples being directly above and below each other, all the way to soil core. Samples that are missing, either due to low DNA concentrations or lack of root/nodule are empty white spaces. ASVs that comprise less than 1% of relative abundance in each sample are grouped together at the bottom of each box.

## Discussion

Understanding the decline of partner quality in microbial mutualists requires integrative approaches that combine the evolutionary conclusions that can be made from culture-based inoculation experiments with the ecological context provided by community sequencing approaches. Here, we reveal that both ecological and evolutionary processes jointly contribute to the breakdown of the agriculturally vital legume–rhizobium mutualism. After 33 years of N-fertilization*,* a key clover mutualist, *R. leguminosarum* ASV-E, was not only more likely to contain a poor-quality pSym (evolutionary change), but entire rhizobial communities shifted such that ASV-E significantly decreased in abundance under long-term N-fertilization (ecological change). Together, the outcomes of these processes limit the growth of clover inoculated with microbial communities from long-term N fertilized plots. To add to the ecological complexity of this system, ASV-E was only one member of a diverse rhizobial community in soil and may be outcompeted by these other rhizobia under N-addition. We discuss these major results below.

### Integrative culture-dependent and culture-independent approaches show that ecology and evolution contribute to mutualism decline

In previous work on the breakdown of the legume–*Rhizobium* mutualism, we used a relatively small collection of ~60 *R. leguminosarum* genospecies E (gsE) and gsB isolates, all isolated from clover nodules, to conclude that N-addition had caused evolutionary changes within the rhizobial pSyms, resulting in poor partner quality for host plants [38–40]. Here, by inoculating clover plants with soil slurries taken from N-evolved plots 15 years after our initial sampling and leveraging full-length amplicon sequencing of chromosomal and plasmid ASVs, we are able to go beyond this previous culture-dependent work to tease apart the concurrent ecological and evolutionary changes underlying mutualism decline. Not only could we differentiate between closely related *Rhizobium* ASVs at the chromosomal level, unpacking genus-level effects to resolve the community shifts beyond our focal ASVs, we were able to independently demonstrate that the shifts in frequency among the three known pSym subtypes persist in nature long after our initial sampling.

Convergence across methods and timepoints both validates that our earlier culture-dependent work captured ecologically meaningful variation, and also newly demonstrates that these pSym variants have persisted over a timescale during which soil communities will have turned over many times. First, our replication allows us to speak to a recurring tension in microbial ecology over whether culture-independent and culture-dependent approaches reach similar conclusions [53, 54], suggesting that, at least for nodule symbionts, the answer is yes. Second, multi-decade monitoring programs have been instrumental in documenting evolutionary change and confirming its persistence in macroorganisms (e.g. Trinidadian guppies [55, 56], Darwin’s finches [57]) but such long-term resampling of microbial communities from nature is exceedingly rare, with long-term work more commonly conducted in laboratories [58]. A few salient examples from nature exist, such as the repeated resampling of stable transconjugants from *Mesorhizobium* associated with *Lotus corniculatus* [59, 60] and the aquatic microbial sampling over 2 decades in a northern temperate lake [23]*.* Here, we add to that body of literature, concluding that the persistent shifts in the frequencies of clover-associated *Rhizobium* pSyms represent stable evolutionary outcomes rather than transient community fluctuations, with N enrichment as a persistent selective force reshaping the symbiotic qualities of rhizobia.

Our approach allowed us to demonstrate that the biomass effects of ecological changes in N-addition soils (decline in ASV-E due to shifts in the soil *Rhizobium* communities and resulting lack of nodulation) were of similar magnitude to the evolutionary changes (rise in frequency of low-quality pSyms). This suggests that, within the legume–rhizobia mutualism, community-scale access to partners is just as important as within-species partner quality. Though past empirical work has shown the importance of symbiont abundance or diversity on host health [61, 62], few studies, if any, compare the magnitude of these effects to within-species evolution of symbiont partner quality. Our work also has interesting implications when considering ways to reverse mutualism breakdown, suggesting that simply replenishing the reservoir of mutualists (even low-quality ones) may improve plant growth. Yet, if the declines in ASV-E result from it being outcompeted by other rhizobial ASVs in soil (ASV-E declines were concomitant with increases in the abundance of other rhizobial ASVs—many of which were not abundant within clover nodules), then supplementing ASV-E into soil may not be effective if the soil community has been restructured in ways that would continue to competitively exclude re-introduced ASV-E [63]. Indeed, because our common garden design captures a snapshot of these communities after 33 years of N-fertilization, we document the outcome of ecological and evolutionary change rather than the field processes that produced it. Multiple mechanisms could have contributed to the clover declines that have been measured in the field [64] and the patterns we observe here, including reduced plant sanctioning of poor symbionts [11, 65], competitive exclusion of clover by non-legumes that are better at using free N [66], or the competitive rhizobial dynamics suggested above. Disentangling their relative contributions remains an important direction for future work.

Though resolving ecological and evolutionary processes in microbial communities is vital, because both can separately and jointly change ecosystems and their functioning throughout time [67, 68], our work highlights how this endeavor is still limited by methodological and taxonomic constraints [22, 23]. For example, the increased read length and high sample coverage provided by PacBio full-length 16S rRNA gene sequencing allowed us to make ecological conclusions about mutualism breakdown (ASV-E decreasing in abundance) at the single-ASV level, because the *Rhizobium* genus did not respond in the same way to N-addition as ASV-E. Thus short-read amplicon sequencing, with resolution at a broader taxonomic level, would have missed this finer-scale change [27, 69]. Even so, our methods leave open questions about within-species evolutionary dynamics. We called our top nodule sequences ASV-E and ASV-B due to the fact that they were perfect full-length matches to *R. leguminosarum* gsE and gsB, the two genospecies we have previously cultured from KBS [40]. However, because the 16S rRNA gene of both genospecies is identical to the 16S rRNA gene of other genospecies [43], we are unable to confidently conclude that these ASVs truly represent one taxon. Thus, though we have demonstrated larger-scale ecological change here by showing ASV-E decreasing relative to other diverse *Rhizobium* ASVs, there may be additional change occurring within the ASV itself (different alleles of gsE or other closely related taxa increasing or decreasing under selection), which we were unable to resolve using our current methods. By examining pSym allele frequencies using a previously identified gsE marker gene, we revealed evolutionary change consistent with selection on gsE pSyms over time. Confirming that these pSym variants are specifically carried by ASV-E, rather than distributed throughout the *Rhizobium* community, requires chromosome-plasmid linkage data such as from Hi-C metagenomic sequencing. These difficulties are not unique to our system; in a 20-year freshwater metagenome time series, within-species evolutionary dynamics were also challenging to differentiate from between-species ecological dynamics, which the authors attributed both to technological limitations of sequencing and unclear taxonomic delineations [23].

### Full-length 16S rRNA gene sequencing resolves rhizobial diversity beyond the limits of database taxonomy

Microbial taxonomy is constantly in flux: reshaped by additional sampling [69, 70], inconsistent nomenclature [71, 72], and variable outputs between different analytical tools and pipelines [73]. Here, even though we restricted ASVs to the combined genus *Allo/Neo/ Para/Rhizobium* (ANPR) within the family *Rhizobiaceae* in SILVA, we recovered ASVs spanning across the family, including ASVs which were part of different, SILVA-defined genera than the ANPR genus (such as *Shinella*). The magnitude of this issue was substantial: less than half of the ASVs identified as ANPR by SILVA truly placed within one of the four ANPR genera, with only ~25% overall in the genus *Rhizobium* s.l*.* This likely reflects the fact that many species formerly known as *Rhizobium* are yet to be renamed in public databases such as SILVA even after being reclassified [42, 44, 71, 73]. Despite this, our family-level phylogeny clearly resolved *Rhizobium* s.l. distinctly from other genera in *Rhizobiaceae,* likely due to both increasing numbers of high-quality references, as well as a significant diversity of ASVs, which we could identify due to our sequencing resolution. Our data demonstrate that, even as genera themselves continue to be revised, full-length 16S rRNA contains the resolution for genus-level inference when paired with current reference data. As such, we believe that future researchers using full-length 16S rRNA gene sequencing may benefit from classifying ASVs phylogenetically against curated references, rather than relying solely on large-scale database classifiers, to gain the most possible resolution from the data [74, 75].

Our data also highlight how inconsistent taxonomic labels may obscure the underlying biological processes driving community change. For example, *Rhizobium* is a genus that was originally defined by its function: N-fixing, legume-associated, root nodule bacteria [76]; yet, in our system, plant compartment explained the largest proportion of ANPR ASV variation, with 73% of our 172 ASVs never detected in nodules at all. Also, many of our ASVs, though changing in abundance throughout compartments and under N-addition, were broadly present throughout all compartments and treatments. Though multiple studies have shown rhizobia upregulated in the rhizospheres of non-legumes [77–79], as well as soil [70], these data have typically resolved rhizobial taxa at family or genus level, leaving open questions about the presence or abundance of specific symbiont clades, such as clover-nodulating *Rhizobium*.

Though our secondary classification has helped resolve taxonomic identity, the majority of the references we used were nodule isolates, whereas our ASVs were primarily found in the soil and rhizosphere. Our data support the idea that the rhizobia are a flexible, plant-associated group—with nodulation and N-fixation representing just one important life history strategy among potentially many other strategies, such as general plant growth promotion [80] through secretion of compounds like lipo-chitooligosaccharides (Nod factors) [81], or potential denitrification (shown in *Bradyrhizobium* [82]). Nevertheless, it is clear that the underlying chromosomal and plasmid diversity of rhizobia remains under-sampled. Additional work integrating metagenomics, long-read amplicon sequencing, and transcriptomics will be key to understanding the distribution and possible functions of rhizobia across plant taxa and soil environments.

## Conclusions

To protect essential mutualisms in a time of rapid environmental change, we require a holistic understanding of both ecological and evolutionary responses. Here, we found that the breakdown of legume–rhizobium mutualism under anthropogenic change was attributable to ecological and evolutionary effects of similar magnitude. It is likely that other vital microbial mutualisms, e.g. soil-forming lichens, reef-building corals, or mycorrhizal networks, are impacted in similarly complex ways by human-caused change, and will require integrated approaches to quantify processes occurring across community, population, and genetic scales.

## Supplementary Material

supplementary_materials_wrag228

## Data Availability

Processed, unfiltered amplicon data (16S rRNA and *nodA*) can be found on Zenodo (DOI: 10.5281/zenodo.17345393). Raw plant data can be found on Zenodo (DOI: http://dx.doi.org/10.5281/zenodo.17410893). All scripts can be found on Zenodo (DOI: http://dx.doi.org/10.5281/zenodo.17410935). Raw sequences are available on NCBI (PRJNA1338501).
